# Reaching older people with PA delivered in football clubs: the reach, adoption and implementation characteristics of the Extra Time Programme

**DOI:** 10.1186/s12889-015-1560-5

**Published:** 2015-03-05

**Authors:** Daniel Parnell, Andy Pringle, Jim McKenna, Stephen Zwolinsky, Zoe Rutherford, Jackie Hargreaves, Lizzie Trotter, Michael Rigby, David Richardson

**Affiliations:** Centre for Active Lifestyles, Institute of Sport, PA and Leisure, Leeds Beckett University, Leeds, UK; Independent Researcher, Football Foundation, Whittington House, 19-30 Alfred Place, London, UK; Football Foundation, Whittington House, 19-30 Alfred Place, London, UK; The Football Exchange, Research Institute for Sport and Exercise Sciences, Liverpool John Moores University, Liverpool, UK

**Keywords:** Football, Physical activity, Older adults

## Abstract

**Background:**

Older adults (OA) represent a core priority group for physical activity and Public Health policy. As a result, significant interest is placed on how to optimise adherence to interventions promoting these approaches. Extra Time (ET) is an example of a national programme of physical activity interventions delivered in professional football clubs for OA aged 55+ years. This paper aims to examine the outcomes from ET, and unpick the processes by which these outcomes were achieved.

**Methods:**

This paper represents a secondary analysis of data collected during the evaluation of ET. From the 985 OA reached by ET, n=486 adopted the programme and completed post-intervention surveys (typically 12 weeks). We also draw on interview data with 18 ET participants, and 7 staff who delivered the programme. Data were subject to thematic analysis to generate overarching and sub themes.

**Results:**

Of the 486 participants, the majority 95%, (n= 462) were White British and 59.7% (n=290) were female. Most adopters (65.4%/n=318) had not participated in previous interventions in the host clubs. Social interaction was the most frequently reported benefit of participation (77.2%, n=375). While the reach of the club badge was important in letting people know about the programme, further work enhanced adoption and satisfaction. These factors included (i) listening to participants, (ii) delivering a flexible age-appropriate programme of diverse physical and social activities, (iii) offering activities which satisfy energy drives and needs for learning and (iv) extensive opportunities for social engagement.

**Conclusions:**

Findings emerging from this study indicate that physical activity and health interventions delivered through professional football clubs can be effective for engaging OA.

## Background

Older adults (OA) represent a diverse group of individuals with varied and complex health profiles and needs [1]. For that reason, they are a priority for policy and guidance in relation to physical activity (PA) and Public Health [1,2]. Consequently, to optimise adherence researchers need to investigate how to effectively design and deliver such approaches [3]. This study explores the initial impact and implementation of a national programme of PA interventions for OA delivered in/by professional football clubs.

Evidence supporting the role of PA for better health is well established. PA can play a powerful role in preventing and/or managing 22 different conditions [4]. To achieve these benefits, contemporary guidelines [2] recommend that OA accumulate at least 150 minutes of moderate intensity PA per-week. This equates to approximately 30 minutes of moderate PA (i.e. brisk walking) on most days of the week, which can be achieved in bouts of 10 minutes or more [5]. Alternatively, for OA who are habitually physically active these benefits can be gained through 75 minutes of vigorous intensity PA (i.e. running or cycling), or a mixture of both moderate and vigorous PA. Further, activities that develop muscle strength and flexibility are also recommended [2]. OA should participate in these activities on at least two days a week. Moreover, OA should reduce the amount of time they spend engaging in sedentary activities (i.e. prolonged sitting) [2,6].

PA can also maintain musculoskeletal strength and improve endurance and flexibility [2]. These assets are all important for functional daily living [1]. Further, PA can positively impact mental wellbeing, in can also improve psychological factors including, self-perceptions and mood [7], as well as reducing anxiety and stress in OA [8,9]. Although social isolation and loneliness are not an inevitable outcome of old age, there is a strong belief that these conditions can occur as a consequence of life events associated with the ageing process [10]. As such, social interaction, social connectivity and social support outcomes resulting from regular PA participation [11] take on an added importance. PA can also reduce demand on Public Health services, predict survival [12], and help to reduce both unplanned hospital admissions and prescriptions for OA over 70s [13].

While evidence on the impact of PA for substantial health improvement is convincing [2,4], PA levels remain low. For example, in the UK only 20% of men aged 65–74 years, and 10% of men aged 75+ years met recommended guidelines. In women, these figures are worse; approximately 17% of women aged 65–74 years and 6% of women aged 75+ years meet guidelines [14]. Regarding ethnicity the picture is even more gloomy [15], for example, fewer than one in 10 South Asians aged 55+ years meet the PA guidelines [16].

The reasons for low PA participation among OA relates to diverse multi-level determinants [1]. For example, at the *individual level* participation can be impeded by perceived and actual barriers, including physical condition, fears over medical status and poor self-image [15]. Further, self-efficacy [1] has been identified as being an important factor both for the initiation and the maintenance of PA [17]. At a *programme level*, key factors include the timing and type of available PA opportunities [1]. At the *environmental level* factors can include social engagement [18] weather and neighbourhood characteristics [1].

OA have previously been recruited to community PA interventions through a range of recommended approaches (i.e. exercise classes, exercise referral programmes and walking groups) [19,20]. A less common, yet clearly effective approach, involves delivering PA programmes through professional sporting clubs [21]. These programmes are typically aimed at advancing the physical and social health of adult populations [22,23]. Even though such approaches may initially appear unsuitable for recruiting OA deemed uninterested in sport and/or PA, they have been successfully deployed and confirmed as important channels for delivering health improvement interventions to otherwise underserved groups [22,24-26].

Since professional football clearly connects with groups unreached by conventional health approaches, it may create an important pathway for health improvement among these groups [23,25,27,28]. Recently, Hunt and colleagues [29] achieved clinically significant weight loss in adult men attending a programme delivered in Scottish professional football clubs. In English Premier League football clubs, adult males - including men aged 55+ − demonstrated statistically significant improvements in an array of lifestyle behaviours within an initial intervention period [30]. Another, pilot, programme delivered to adults aged 55+ in an English football club for men and women [22] showed that while women reported ‘higher’ self-rated health than men, there were no gender difference in attendance; each participated in 75% of sessions. These findings support the potential of professional football clubs as ‘community hubs’ for strongly engaging OA in health in improvement activities.

### Purpose

To address this potential we examined the implementation and outcomes of Extra Time (ET), a national programme of interventions delivered in/by Premier and Football League clubs for OA aged 55+ years [31]. First, this paper addresses the self-report outcomes of ET. Second, qualitative analysis examines how these outcomes were achieved.

## Methods

### Intervention context

Funded by the Football Foundation, Comic Relief and Age UK, ET centred on PA based social inclusion/health-improvement activities and were delivered free of charge over two-years [31]. Beginning with 15 Premier League and Football League clubs, and always led by a designated community practitioner, each club was awarded approximately £10,000 to deliver their ET intervention. In year two, 13 clubs remained, supplemented by seven clubs starting new interventions. These interventions varied by club as seen in other football-led health improvement programmes [30].

ET’s community practitioners typically possessed - as a minimum - a National Governing Body Level 2 sport qualification. Even though they required experience of delivering community partnerships on similar health interventions at football clubs [23], few were experienced in working with OA. In response, the Football Foundation and Age UK combined to design and deliver specific education on promoting PA in OA (a toolkit now shows how to deliver ‘effective’ ET interventions) [31].

The overall national direction of ET was established by discussions, focus groups, and conversation with OA and key workers, as recommended by Public Health guidance [1]. Then each club framed these findings against local concerns to refine their provision and approach. The national programme of ET was delivered through club-based interventions. Consistent with previous work, interventions typically involved weekly classes and groups [23-25]. These provided physical and social activities linked to health-improvement for OA. The classes continually evolved in response to on-going informal conversations and discussions with participants, their supporters and key workers and through formal discussions with participants and key stakeholders [30].

To address local health priorities, clubs were encouraged to work within their local strategic context to achieve health improvement priorities. For example, the Football Foundation directed clubs to develop partnerships, which aligned to local Joint Strategic Needs Assessments (JSNAs) [32]. Clubs were also required to link project objectives to their respective Local Area Agreement (LAA) [33]. To achieve this end, practitioners formed local steering groups incorporating OA, delivery agents and strategic partners involved in undertaking JSNAs and LAAs to inform the decision-making processes. Table 1 shows selected examples of the types of partnership, activities and composition of the steering groups.

**Table 1:** **Examples of local steering group partnerships**

***Football club***	***Partners***	***Nature of their role***
Bristol Rovers	*Alzheimer’s Society*	Providing initial and on-going advice about working with OA and the referral of OA.
	*South Gloucester Council*	Financial support along with advice on the local strategic fit and how the project fitted with their priorities.
Colchester United	*Age Concern Colchester*	Identifying five day centres to host the PA delivery and specific guidance the delivery of PA for OA.
	*Colchester Borough Council*	Providing a broad knowledge of local issues and arranging additional support/access to services e.g. Health visitors.
Rotherham United	*NHS Rotherham*	To lead in the development of the project, build capacity and provide continued support and guidance.
	*Age Concern Rotherham*	To provide and share information relevant for delivering a bespoke OA intervention.
Scunthorpe United	*NHS North Lincolnshire*	To play a key role in developing the pilot intervention and assist in gaining access too hard to reach groups.
	*N. Lincolnshire Council*	Providing advice on intervention development and OA referrals to the ET project.
QPR	*Open Age*	Provide advice and recommend delivery strategies for OA

Using domain-specific frameworks for intervention mapping and design [30], Table 2 shows the key focus and key implementation characteristics of participating clubs. Generally, activities consisted of 2 hour-long weekly classes and groups involving physical and social activities delivered free to participants [34]. A broad menu of PA opportunities were offered, including exercise to music, indoor bowls, cricket, new age curling, walking football, alongside traditional board games, bingo, table tennis, zumba and skittles, as seen elsewhere [22].

**Table 2:** **Extra time programme by club based intervention, participants and design**

	**Target group**										**Intervention design/content**								
															**Activity**				
**Club**	**Gender**		**Age**				**Ethnicity**		**Disability**		**Mode**		**Venue for classes**		**Physical activity**			**Social activity**	**Health education**
	**m**	**f**	**50-59**	**60-69**	**70-79**	**80+**	**WB**	**BME**	**YES**	**No**	**Match day**	**Class**	**Stadia/club facility**	**Community venue**	**Foot ball**	**Gym**	**Other**	**Games**	
1*	✓	✓	✓	✓	✓	✓	✓	✓	✓	✓	✓	✓	✓	✓	✓			✓	✓
2**	✓	✓	✓	✓	✓		✓	✓		✓		✓	✓	✓	✓			✓	
3*	✓	✓	✓	✓	✓	✓	✓					✓	✓	✓	✓	✓		✓	✓
4**	✓	✓	✓	✓	✓		✓	✓	✓	✓	✓	✓	✓	✓	✓		✓	✓	
5**	✓	✓					✓					✓	✓	✓			✓	✓	✓
6**	✓	✓	✓	✓	✓	✓	✓		✓	✓	✓	✓	✓	✓			✓	✓	
7*	✓	✓	✓	✓	✓		✓			✓		✓	✓	✓	✓	✓	✓	✓	✓
8*	✓	✓	✓	✓	✓	✓	✓	✓	✓	✓	✓	✓	✓		✓			✓	✓
9*	✓	✓	✓	✓	✓	✓	✓		✓	✓	✓	✓	✓	✓	✓		✓	✓	✓
10**	✓	✓	✓	✓	✓		✓	✓		✓		✓	✓	✓			✓	✓	
11**	✓	✓	✓	✓	✓	✓	✓		✓	✓	✓	✓	✓		✓	✓	✓	✓	✓
12**	✓	✓	✓	✓	✓		✓		✓	✓	✓	✓	✓	✓	✓		✓	✓	✓
13*	✓	✓	✓	✓	✓	✓	✓		✓	✓		✓	✓		✓		✓	✓	✓
14*	✓	✓	✓	✓	✓		✓	✓	✓	✓		✓	✓				✓	✓	
15**	✓	✓	✓	✓	✓	✓	✓	✓	✓	✓		✓	✓	✓	✓		✓	✓	✓
16**	✓	✓	✓	✓	✓	✓	✓					✓		✓				✓	
17**	✓	✓	✓	✓	✓	✓	✓	✓	✓	✓		✓	✓	✓			✓	✓	✓
18**	✓	✓	✓	✓	✓	✓	✓					✓		✓	✓		✓	✓	
19**	✓	✓	✓	✓	✓		✓	✓	✓	✓		✓	✓	✓	✓		✓✓	✓	✓
20**	✓	✓	✓	✓	✓	✓	✓	✓	✓	✓	✓	✓	✓		✓		✓	✓	✓

Key: *Football club has been funded from year one, **Football club has been funded from year two, WB = White British, BME = Black, Minority, Ethnic.

Endorsing the social component of PA, classes provided participants with an opportunity to socialise before, during and after sessions. Light refreshments were often provided after activities, while more formal, organised social events including day trips were also provided. Activities were commonly delivered within the stadia or club facilities. While outreach work has been recommended [35], the football club and stadia - which are trusted, iconic and community embedded facilities - were most commonly used [23].

Posters, flyers, existing contacts and club media channels, such as match day programmes, the club website and magazines were used to recruit OA to ET. Advertisements were also placed in the local press and free newspapers [36]. In some clubs, outreach work was undertaken where OA congregated (including local health centres) and where community leaders were willing to help raise awareness of the programme [24,30]. ‘Word of mouth’, whereby participants would tell their friends, was considered central to recruitment [30].

In line with recent recommendations [37], this study reports the reach, adoption profiles and key implementation characteristics of the ET programme, which are underpinned by the REAIM framework [38]. The REAIM framework has been used to evaluate public health interventions, including football-based approaches. REAIM not only provides a comprehensive structure for assessing the impact of interventions across the behavioural change continuum (Reach, Adoption and Maintenance), but also the processes by which interventions have an impact (Effectiveness) [39].

#### Methods of data capture

Ethical approval was obtained from the Local Research Ethics Committee of the Carnegie Faculty at Leeds Beckett University. This permitted secondary analysis of the original evaluation data collected by the Football Foundation between May 2008 and July 2010. Informed consent was collected from participants in the original service level evaluation. Training to undertake the original evaluation was delivered by the Football Foundation, as part of a national training event prior to project commencement. Self-reported data was captured from OA at pre-intervention by club-based community practitioners, typically at first point of contact, i.e. ET induction. This was administered via a survey-based protocol which addressed participants’ (i) demographics, (ii) experience within the intervention, (iii) self-rated indices of health, fitness and PA, (iv) past engagement with the host club and (v) the features that drew them into ET.

To identify the key intervention implementation characteristics, the research aimed to understand the perspectives of both participants and key stakeholders. Adopting a convenience sampling system, researchers conducted semi-structured interviews with participants and key ET intervention staff. Interview schedules were developed with consideration of previous health improvement interventions and attention to the Reach and Adoption elements of the REAIM framework [38]. These interviews were based on approaches that have been effective in other work exploring the experience of participants in PA-led health improvement interventions [40], including benefits, barriers, facilitators and promotion, which have their origins in research performed with OA and PA [41].

### Statistical analysis

Descriptive statistics were undertaken to highlight the socio demographic profiles and characteristics of participants and to help establish the total Reach and Adoption of ET [30]. As data were not normally distributed, Mann–Whitney (U) tests tested for differences between two conditions (age category and gender), Kruskal-Wallis tests (H) measured differences between several independent groups (self-reported benefits and physical activity) and the Jonckheere-Terpstra test (J) identified ordered patterns in the medians of comparison groups (self-reported benefits and physical activity). All analyses were conducted using SPSS for windows version 21.0.

### Qualitative data analysis

Adopting a convenience sampling system [42], a researcher, independent of the ET programme (LT), carried out semi-structured interviews with 18 participants from five clubs and with seven key ET intervention staff. These interviews were undertaken between August-October 2010. After 18 interviews with participants, recurring comments were emerging and the researcher felt that data saturation had been achieved with respect to the aims of the evaluation. All interviews were digitally recorded and transcribed verbatim; a pseudonym was used to identify each participant. To address both inductive and deductive content, the analysis was undertaken in two stages. The first stage involved identifying the obvious commentary relating to either Reach or Adoption in each transcript. All the responses for Reach and Adoption were pooled to provide a single account for each factor.

The second stage involved new researchers (DP, DR) returning to the original files and undertaking a thematic analysis [30,43]. The process of thematic analysis is split in to six phases, (i) familiarisation with the data, (ii) generating initial codes, (iii) searching for themes, (iv) reviewing themes, (v) defining and naming themes and finally once there is a set of fully worked out themed (vi) writing up [43]. However, this type of analysis adopts a flexible methodology, researchers don’t purely move from one phase to the next. Instead, it is a more recursive practice where the route is back and forth as required.

The themes we now report were consistent (a) *within* a stage of analysis and (b) *across* the stages of analysis. This provides a high level of cross triangulation. This consolidation of data was established through discussion within group meetings between all co-authors. It is important to note that our purpose in presenting the contextual voice using verbatim extracts is to allow the reader to get to “know well” a few participants, as opposed to knowing little about many. In a philosophical sense, we do not claim that the narrative themes that emerged are generalizable to all. We also contend that researchers and readers of this paper should appreciate that all phenomena should be judged as time- and context- specific [44].

## Results

### Socio-demographic profile of ET participants

In total N=985 OA were reached by the ET programme, as evidenced by completion of the baseline survey. In terms of adoption and adherence to the evaluation, n = 486 participants attended the ET projects and provided follow-up data. The following analysis is based on this sample of 486.

Table 3 shows the socio-demographic characteristics of ET participants. The intervention engaged more females (59.7%) than males (40.3%) males, and although it was aimed at participants aged 55+, some were younger. Overall, 5.3% of participants were 50–54 years old, 59.9% were 55–74 and 34.8% were 75+. Female participants were significantly older (*Mdn* = 70–74 years) than males (*Mdn* = 65–69 years), U = 21778, z = −4.445, *p* < .001, *r* = −0.20. White British participants represented 95.1% of participants, while 9.7% were registered as disabled.

**Table 3:** **Socio demographic characteristics of respondents**

		***Percentage of participants (n)***
***Socio demographics***		
Gender (n = 486)	*Male*	40.3 (196)
	*Female*	59.7 (290)
Age (n = 486)	*50-54*	5.3 (26)
	*55-59*	4.5 (22)
	*60-64*	21.6 (105)
	*65-69*	18.9 (92)
	*70-74*	14.8 (72)
	*75-79*	14.0 (68)
	*80+*	20.8 (101)
Ethnicity (n = 486)	*White British*	95.1 (462)
	*Black/Black British*	2.3 (11)
	*Asian/Asian British*	0.4 (2)
	*Chinese*	0.2 (1)
	*Mixed*	0.8 (4)
	*Other*	1.2 (6)
Registered disability (n = 486)	*Yes*	9.7 (47)
	*No*	90.3 (439)

### Club affiliation of participants

Participants came from 16 different professional football clubs; Rochdale (17.7%), Blackburn Rovers (11.3%) and Colchester United (10.9%) were the largest individual recruiters. Most adopters (i.e., participants providing data at pre and/or post intervention on their health related behaviour) - 65.4% - had not previously participated in events or programmes held by the host club. A greater proportion - 80.2% - reported that the link to a professional football club made ET more appealing because of the sense of community it created. This single factor was more important than being a fan (24.3%) and the facilities available (16.9%). Overall only 7.8% of participants became fans of the host club after engaging with ET.

### Self-reported benefits of adopting ET

The most frequently self-reported benefits of engaging with ET were making friends and having fun (77.2%, n = 375), feeling healthier (55.2%, n = 249) and feeling happier with life (35.6%, n = 173). Moreover, PA participation significantly increased the proportions of participants who reported feeling healthy (H [3] = 31.834, *p* < .001) and fitter (H [3] = 12.538, *p* < 0.05). Jonckheere’s test revealed a significant trend in the data; as the frequency of physical activity increased participants reported feeling healthier (J = 38317, z = 4.196, r = −0.19) and fitter (J = 35932, z = 2.564, r = −0.12).

### Process evaluation

Analysis of the semi-structured interviews identified a number of key themes. These are mapped against the Reach and Adoption elements of the RE-AIM framework [38,39] alongside considerations for promoting health improvement. The following results represent the summary of our two stage analysis and interpretation (Table 4).

**Table 4:** **Process evaluation themes and sub-themes**

***Overarching themes***	***Thematic sub themes***
*Reach*	The appeal of the football club
	Building on existing, and developing new community networks
	Spreading the word
	Strategies to build interest in ET
*Adoption*	Increasing opportunities to play football
	Enjoyable activities
	Enriching opportunities to socialise

### Reach

#### The appeal of the football club

Participants reported that the ‘football club’s badge’ helped to reach OA and had a positive influence on participants’ interest in the programme:

*“There are places you can do exercise, but going to the football club for exercise is different. It’s special and not something I have seen before. I had to give it a go”.* (Lenny – participant)

*“I have loved the club all my life and used to go when I was younger. It’s fantastic to be back here”.* (Barry – participant)

While ET was gender-neutral, engaging older men into health and activity interventions has previously been shown to be problematic. Yet, a number of wives and partners reported how the reach of football/football club helped facilitate the involvement of their male partners:

*“The football club has achieved more than I could have. There’s no-way my husband would have done line-dancing if I’d suggested it!”* (Joyce – participant)

ET intervention staff also picked up on the potential appeal of the football club:

*“Many of these people are local to the community and are often supporters of the club. This certainly helped me, as the club captured their imagination and created an excitement for both those* [local agencies] *and the participants. Something you don’t get without the brand of the club”.* (Dan – intervention staff)

### Building on existing, and developing new, community networks

Successful recruitment often involved getting ‘buy in’ from the participants and attracting those who had OA caring responsibilities. One intervention lead reported:

*“Working with older adults in various setting from care homes to sheltered accommodation is different to working with the ‘average Joe’ we are used to* [children, men, women], *who walk in ‘off the street’ to one of our projects. We had to get the buy-in from managers of care homes and care assistants*”. (Peter – intervention staff)

Understanding this, some of the club staff adopted outreach activities to highlight the availability and provision of ET. These staff often reported the importance of utilising their existing networks to facilitate reach:

*“We had done some past engagement with care homes, which meant we could market our ET intervention directly to the people we needed to engage with. We managed to meet every care home manager in the North West* [of England] *in one meeting, as the director* [of the care home business] *was a fan of our club. We got their buy-in and the promotion needed to recruit*”. (Simon – project staff)

### Spreading the word

One of the most powerful ways of connecting with potential participants was through word of mouth; the social advantages being advocated in these exchanges were important:

*“One of the gents* [ET intervention staff] *came to our group where we play dominoes and told us about this at session at the club. We jumped at the chance to meet new people and try out a few of the activities”.* (Gary – participant)

*“I just heard through friends. It’s how it works. I have told people who have started to come too”.* (Tosh – participant)

For some clubs, it was necessary to develop new networks in the local community before they could begin to reach OA:

*“As a club, we hadn’t worked with the elderly before. My usual contacts within schools wouldn’t be any use. I had to work across our organisation to reach-out to our existing contacts in the community who worked with the elderly and in many cases, develop a new of set contacts. We also had information the match-day programme, but our greatest success was through our new contacts, who became partners and really important to our success in getting access to the elderly*”. (Peter – intervention staff)

Outreach approaches that involved linking with key community workers were helpful for recruiting potential participants were unlikely to encounter promotional events:

“*I don’t get to the match these days, so would never of heard of the project was it not for Jason* [community worker from the NHS]”. (Mark – participant)

*“We don’t leave the sheltered the accommodation. If people don’t come to us or if Mo* [carer within the sheltered accommodation] *doesn’t get people in, then we don’t get to hear about it”.* (Dennis – participant)

ET staff aspired to engage with a range of service providers. In the understanding that many OA are living independently within the community, links with older people’s services offered an important route to connect with potential recruits:

*“I heard about ET through the Alzheimer’s Society. I thought ‘Well, I like company so I thought what a good idea!”* (Pam – participant).

### Strategies to build interest in ET

Programme staff understood that a participant’s reluctance to take the first steps into ET would be multifaceted. Therefore, following awareness raising sessions undertaken at key settings, potential participants were offered the opportunity to attend a game. This was part of a staged approach, beginning with getting to know the club and from there, to attract them to the programme.

*“We provide free tickets to those that haven’t been the match before or for some time”.* (Mark – intervention staff)

*“Going to the game is a great outing. We get to support the club and soak up the atmosphere. I haven’t been the game for years and I loved it”.* (Martin –participant)

### Adoption

#### Increasing opportunities to play football

Adoption is a key consideration for the development of effective interventions. Therefore, it is important to better understand the factors that encourage and support adoption among this group. Once individuals knew about the programme, a broader range of factors helped to encourage individuals to take part in activities. For a number of individuals playing football was important:

*“I shall keep playing football as long as I can. I enjoy each game I play even though some of the 'younger' players are a lot faster than me. That’s why I come”.* (Don – participant)

Recognising that every participant would not be able to play - let alone be interested in - football, a diverse range of physical and social activities were offered:

*“When Roz* [ET intervention staff] *from Tottenham came to our accommodation; she offered us lots of activities to choose from and also when we would like to do the activities. This was important to us*”. (Irene – participant)

### Flexible delivery for a variety of PA options and activities

Providing a flexible menu of activities and timings was considered an important design characteristic. This was especially true for those individuals who self-assessed as having poor fitness and were diagnosed with ill health:

*“I wasn’t a real big football fan and haven’t played it here at all. We have so many different activities in place and available that many would prefer; from dancing, day-trips, to horse riding. Things I never thought I would be doing when I joined!”* (Lenny - participant)

### Enjoyable activities

Enjoyment and exposure to new activities generated social engagement and often helped to promote engagement with PA:

*“I miss playing football, but we have plenty of fun with indoor curling. Something I never thought I would like or even try!”* (John – participant)

*“We have such fun trying new activities at the club or on our little trips*”. (Brenda - participant)

*“The boys that run our sessions are always full of energy and make everything fun. It doesn’t matter whether we are baking something, telling stories or playing board games!”* (Maureen – participant)

### Enriching opportunities to socialise

The social aspect of the programme was an essential component supporting involvement and retention, which was often likened to rejuvenation:

*“It’s the social side I have missed. That’s what I miss since I stopped playing football too – the banter. Now I got it back again. We act like teenagers sometimes!”* (John - participant)

*“Playing some football is great, but I enjoy the social side of the project, especially the trips away to play matches and to visit other grounds. Some of the other players have become really good friends of mine”*. (Jon – participant)

ET also appealed to a number of couples, who attended together. For one couple, Jenny and Ben, the regular sessions were an important part of their weekly schedule of events and provided an important source of social support, beyond how they supported one another. The opportunity to be with people who handle similar on-going daily challenges, especially a partner’s (especially husband’s) lost cognitive abilities, was important. Carol, an ET deliverer, told their story:

*“For one of our participants, Jenny, a full-time career for her husband. ET gives them both an opportunity to get out the house, have some time apart to mix with others, and do things they wouldn’t normally do. Jenny says there is no stigma at Rovers and they both look forward to the sessions and enjoy them each week. There are six or seven women at the group whose husbands have dementia or Alzheimer’s. They are all at different stages of the disease and the wives share their experiences”.* (Carol – intervention staff)

Jenny concurred; she saw the programme as ‘life changing’:

*“It’s good to know you’re not alone in what you’re going through. I have made one good friend in particular, who I regularly email and speak to on the phone. We have the same outlook and try to provide support to one another. ET has changed our lives – if we can be active then Ben is ok*”. (Jenny –participant)

### Considerations for promoting health improvement

While the programme reflected many of the implementation characteristics previously identified as important for facilitating both reach and participant adoption inevitably there were various learning opportunities:

*“I think when we first came the boys* [intervention staff] *expected us to want to sit and play board games or bingo like proper OAPs* [old aged pensioners]. *We soon changed that and before long we have exercises sessions in the club stands, yoga classes and our favourite, indoor bowls!”* (Sandra – participant)

The challenges associated with engaging profoundly isolated OA remain difficult for even the most experienced practitioners and organisations. Whilst the ET programme successfully engaged a proportion of socially isolated OA, most were already socially active to some degree:

*“We had to work with organisations that had experience and credibility working with older people. Naturally, people already in the system* [engaged in social appointments during their week-to-week routine] *wanted to also attend our sessions”*. (Jason – intervention staff)

ET programme represented an exit route or add-on to existing provision for many organisation and participants. Given that ET was a new programme adopting alternative channels to deliver provision, few football clubs had focussed their attention on offering exit routes once initial funding was gone:

*“We really struggled for exit routes, especially new ones. When we asked our partners, for their exit routes, they explained that we* [i.e., the club through ET] *were the exit route for PA and sport!”* (Don – intervention staff).

## Discussion

The current study aimed to determine the reach, adoption profiles and key implementation characteristics of the ET programme. Our results offer positive preliminary evidence of both the influential processes and the potential for this programme. Results suggest that ET interventions, even allowing that each club had a distinctive profile of delivery and content, were effective in reaching and helping OA engage in physical and social activity. These findings are important given that at the time of writing, and to the best of our knowledge, there are no studies offering such insights into the impact of a multi-site professional football-led health intervention for OA.

### Who adopted the ET programme

ET was especially effective in reaching and recruiting participants of white British decent, across a wide range of age and physical abilities. The programme was equally acceptable for women as well as men, although slightly more females than males attended. Consistent with previous evidence [24], female participants often reported that the programme provided an opportunity to attend with their male partners. This suggests that female partners can have a unique impact on male participation.

It is also important to acknowledge that female partners recognised the ‘leverage’ that being associated with football and a professional football club, represented for their male partners. Their vigilance lead to doing beneficial activities together and generated important opportunities for learning and for meeting new people. That said, the programme was also acceptable to older men, and seems to overcome the strong barriers that had prevented them from engaging with other PA and health interventions [30]. Locating the programme in a setting aligned to men’s interests and networks may have been important [24,25,30].

However, a number of key target groups were under-represented in this study, including individuals from BME backgrounds and/or individuals reporting a disability. While older men from BME groups have been recruited into football-led interventions in the past [30], this often required extensive outreach work. Moreover, there is a large gap in the evidence when considering BME women. South Asian women can often experience social and cultural determinants to participation in PA programmes [1]; ET had a limited impact in overcoming these issues [16,45]. Although the recruitment successes of ET reflect the community partnerships fostered by club staff, greater commitment is needed to building effective partnerships with underserved BME groups. In this instance, it may be that football does not have the same appeal or prominence in these communities.

### What factors extended ET reach among OA?

The ‘reach of the club badge’ helped to establish partnerships that enhanced recruitment [24-26]. This effect was reported beyond those few participants who reported themselves as being fans of the club where they attended ET. That said, this route was never expected to be anything more than limited, even though clubs continued to use their own media channels for communicating about the programme. Notwithstanding their obvious potency for some individuals, our experience was that these channels had only a limited reach into the communities targeted by ET [46].

Instead, an expanded outreach programme was typically undertaken by club staff to foster new and existing partnerships and to work together with agencies routinely providing services for OA. These partnerships often secured access to ‘new’ groups of OA. This was particularly important in ‘spreading the word’ among potential participants [25]; once participants had engaged with ET, they were not shy about ‘spreading the word’ among their networks.

### What factors helped to attract OA to adopt the ET programme?

While the attraction of football and the club created initial interest and reach for some participants, a more specific set of design characteristics attracted people into the programme [24]. Many participants reported that the most attractive aspect of the intervention was the regular opportunity for social support and interaction; this included the opportunity to meet new people. It is likely that few of the programmes would have worked so well without providing these opportunities. Social support is an important known mediator of PA participation, especially for those with limited social connectivity and partners in caring roles [47]. Even the anticipation of experiencing social activities was enough to attract many OA into ET.

Participants also reported that they enjoyed being active through a varied programme of social and physical activities. These activities were offered routinely and for a number of participants weekly routine was central to their attendance. Consistent with previous research [48]. Routine was especially important for those in residential care; complementing the routines of residential homes was important to participants.

National guidance reflects the need for OA to be consulted when designing interventions aimed at them [35] and it is heartening that so many clubs engaged in this process. In ET, this appears to have contributed to strong recruitment and impressive adherence. ET was associated with many humorous, exciting and enjoyable activities and events and this was often a centre-point of interviews. Consistent with this, while football was provided, other physical activities were also offered, supporting the diverse age range and abilities of attendees.

‘Word of mouth’ from friends seemed especially influential in establishing the first steps of adoption, particularly when these were first-hand accounts offered to like-minded individuals about the intervention and its benefits. Although this was often a slow process for enhancing recruitment – far slower than intervention staff would have liked - it reflects that the participants were enjoying their experiences within ET and wanted to share it with others. Typically, ‘word-of-mouth’ provided reassurance to potential participants about returning to PA; these recommendations were powerful in allaying apprehensions about attending an unfamiliar programme [30]. Receiving a recommendation from ‘someone like me’ makes a big difference compared to a similar endorsement from a stranger or someone much younger. Deploying similar approaches may be important for connecting with groups left unreached by ET.

Finally, the actions of the intervention staff (i.e., community practitioners/coaches) who led the activities were central to programme success; they shared a commitment to providing a meaningful, relevant and enjoyable programme of health improvement activities. This has been seen elsewhere [39]. For many staff, ET meant navigating ‘new waters’ for working with OA and they showed how well they converted their learning into successful delivery. This represents not only a success for ET but also shows how useful it was for ET staff to work with community services focused on OA needs. The desire of key staff to learn and refine the implementation helped to ensure that the interventions met the needs and satisfied participants [24].

The ET programme offered a number of points that may help to refine the planning and delivering of future programmes of football-led health improvement with OA. Some participants reported that clubs had initially pitched the menu of physical activities at the ‘less strenuous’ level (i.e., unlikely to engage participants in moderate to vigorous PA), when – perhaps counter-intuitively - a more challenging set of physical activities was required. This finding appears to endorse the reality that OA represent a group with widely varying health needs and physical capacities. It is important, therefore, to consider the volume and intensity of activities on offer, and how they correspond to any participant’s capability. The challenge for delivery staff is to provide activities that have the capacity to be strenuous enough to elicit the physiological changes that some recruits were pursuing, but not so vigorous that the minimal engagement negatively impacts the adherence of others.

Limitations and learning also apply to the evaluation strategy. For example, involving the researchers in this study at the earliest stages of planning may have resulted in a more powerful monitoring framework. In particular, this would have ensured the use of validated items for measuring PA and health impacts, including objective measures. The absence of a specific question in the original evaluation meant that it was not possible to establish how well these adoption profiles indicate new behaviour or if ET simply replaced pre-existing PA. Moreover, conducting interviews with non-completers and non-adopters would have provided different perspectives on the programme and, potentially, identified both barriers and solutions to participation/adoption.

Even though qualitative research typically relies on small sample sizes, this research may have benefited from recruiting a more diverse – and larger - sample of participants to contribute interviews. Limited resources often accompany ‘real world’ evaluations and may have prevented this [48]. Strengths included a partnership model of evaluation, characterised by a strong commitment to work with the deliverers, i.e., community practitioners/intervention staff. The multi-method design incorporating both impact outcomes from participants and providers offers a helpful approach to research design. By working in partnership with evaluators, the clubs have helped to establish a template for subsequent evaluations.

## Conclusion

The rising cost of health and social care for OA represents a major public health issue. This research contributes to the growing literature on football-led health improvement, while extending it to address provision for OA. Results suggest that professional football can effectively reach and recruit male and female OA into a programme of physical and social activity. While the reach of the club badge was important in letting people know about the programme, further work enhanced adoption and satisfaction with the programme. These factors included (i) listening to participants about programme provision, (ii) delivering a flexible age-appropriate programme of diverse physical and social activities, (iii) offering activities which satisfy energy drives and needs for learning, and (iv) extensive opportunities for social engagement. Findings emerging from this study are important for delivering effective health improvement programmes for OA in football settings.

## Abbreviations

OA: Older adults
ET: Extra time
PA: Physical activity
